# Tubercular Deposits in the Bronchial Glands

**Published:** 1843-05

**Authors:** 


*Tubercular Deposits in the Bronchial Glands.*—Drs. Rilliet and Barthez, from their extensive researches into this interesting subject, find, that, by compressing the vena cava superior, tubercular enlargement of the bronchial glands may be followed by—1. Œdema of the face; 2. Dilatation of the veins of the neck; 3. Livor, in a greater or less degree, of the countenance; 4. Hemorrhage into the arachnoidal cavity.

By compressing the pulmonary vessels they occasion—1. Hæmoptysis; 2. Œdema of the lungs.

When they press upon the pneumogastric nerves, they cause—1. Alterations in the pitch and quality of the voice, and cough; 2. Violent fits of coughing, resembling those of hooping-cough; 3. Asthmatical attacks.

The action of enlarged and tubercular bronchial glands on the lungs and bronchi is very remarkable. By compressing the air-passages, they produce—1. Sonorous *râles* of great intensity, very persistent, and of which the quality is sometimes very peculiar; 2. They impede the access of the air, whence follows obscurity in the respiratory murmur, though this sometimes depends on the œdema of the lung, which is consequent on the pressure upon its returning blood-vessels. Sometimes they serve as conductors of sonorous vibrations, from which the following effects ensue:—1. Alterations in the character of the respiratory murmur, the lungs themselves being perfectly healthy, such as prolonged expiration, bronchial respiration, and all the sounds which, in the normal state, must take place in the bronchi, but which do not reach the ear; 2. Great extension of the stethoscopic indications of any particular lesion, as from the one side of the chest to the other.

The observations, of which the above summary is given, were made upon children.—*Lond. and Edin. Med. Journ. Med. Sci.*, Feb. 1843, from *Archiv. Gén. de Méd.*, December, 1842.

